# Metoclopramide to Prevent Pneumonia in Patients With Stroke and a Nasogastric Tube: Data From the PRECIOUS Trial

**DOI:** 10.1161/STROKEAHA.124.047582

**Published:** 2024-08-12

**Authors:** Wouter M. Sluis, Jeroen C. de Jonge, Hendrik Reinink, Lisa J. Woodhouse, Willeke F. Westendorp, Philip M. Bath, Diederik van de Beek, H. Bart van der Worp

**Affiliations:** Department of Neurology and Neurosurgery, Brain Center, University Medical Center Utrecht, Utrecht University, the Netherlands (W.M.S., J.C.d.J., H.R., H.B.v.d.W.).; Stroke Trials Unit, Mental Health and Clinical Neurosciences, School of Medicine, University of Nottingham, United Kingdom (L.J.W., P.M.B.).; Department of Neurology, Amsterdam University Medical Center, Amsterdam Neuroscience, the Netherlands (W.F.W., D.v.d.B.).

**Keywords:** cerebral hemorrhage, humans, ischemic stroke, metoclopramide, pneumonia

## Abstract

**BACKGROUND:**

A randomized trial suggested that treatment with metoclopramide reduces the risk of pneumonia in patients with acute stroke and a nasogastric tube. We assessed whether this finding could be replicated in a post hoc analysis of the randomized PRECIOUS trial (Prevention of Complications to Improve Outcome in Elderly Patients With Acute Stroke).

**METHODS:**

PRECIOUS was an international, 3×2 partial-factorial, randomized controlled, open-label clinical trial with blinded outcome assessment assessing preventive treatment with metoclopramide, paracetamol, and ceftriaxone in patients aged ≥66 years with acute ischemic stroke or intracerebral hemorrhage and a National Institutes of Health Stroke Scale score ≥6. In the present study, we analyzed patients who had a nasogastric tube within 24 hours after randomization. Patients who were allocated to metoclopramide (10 mg TID) were compared with patients who were not. Treatment was started within 24 hours after symptom onset and continued for 4 days or until discharge if earlier. The primary outcome was pneumonia in the first week after stroke. The score on the modified Rankin Scale after 90 days was a secondary outcome and analyzed with ordinal logistic regression.

**RESULTS:**

From April 2016 through June 2022, a total of 1493 patients were enrolled with 1376 included in this analysis, of whom 1185 (86%) had ischemic stroke and 191 (14%) had intracerebral hemorrhage. The first day after randomization, 329 (23.9%) patients had a nasogastric tube, of whom 156 were allocated to metoclopramide and 173 to standard care. Metoclopramide was not associated with a reduction of pneumonia (41.0% versus 35.8%; adjusted odds ratio, 1.35 [95% CI, 0.79–2.30]) or with poor functional outcome (adjusted odds ratio, 1.07 [95% CI, 0.71–1.61]).

**CONCLUSIONS:**

In patients with stroke who had a nasogastric tube shortly after stroke onset, metoclopramide for 4 days did not reduce pneumonia or have an effect on the functional outcome.

About half of the patients have dysphagia in the first days after stroke, hampering food intake and risking undernutrition.^1,2^ Undernutrition is associated with an increased risk of death and poor functional outcome.^3,4^ Early commencement of feeding via a nasogastric tube might reduce the risk of death but has not been proven to increase the chance of a good functional outcome.^5^ On the contrary, early tube feeding may also increase the risk of pneumonia, another strong predictor of a poor functional outcome.^6^ Pneumonia after stroke can be caused by dysfunction of the lower esophageal sphincter after stroke, predisposing patients to reflux of stomach contents and aspiration.^7–9^

It has been hypothesized that the prevention of aspiration pneumonia through improving lower esophageal sphincter tone and gastrokinesis with the dopamine antagonist metoclopramide could improve functional outcome in patients with stroke. In the randomized phase II MAPS trial (Metoclopramide for Avoiding Pneumonia After Stroke Trial), metoclopramide reduced the occurrence of pneumonia from 87% to 27% in patients with stroke and a nasogastric tube and led to a nonstatistically significant reduction in the rate of death at 30 days from 40% to 27%.^10^ The effect on functional outcome was not assessed. A phase III clinical trial to evaluate the effects of metoclopramide in patients with stroke with a nasogastric tube is ongoing (MAPS-2 [The Second Metoclopramide for Avoiding Pneumonia After Stroke Trial], ISRCTN40512746). By contrast, metoclopramide started within 24 hours of stroke onset and continued for 4 days had no effect on the risks of pneumonia and death in elderly patients with stroke with or without a nasogastric tube in the phase III randomized PRECIOUS trial (Prevention of Complications to Improve Outcome in Elderly Patients With Acute Stroke).^11^ In the present study, we assessed whether metoclopramide reduced pneumonia and improved functional outcome in the subgroup of patients included in PRECIOUS who had a nasogastric tube.

## METHODS

### Study Population

This is a post hoc analysis of data from PRECIOUS (ISRCTN82217627), a European, multicenter, 3×2 partial-factorial, randomized controlled, open-label clinical trial with blinded outcome assessment of the preventive use of metoclopramide versus no metoclopramide, ceftriaxone versus no ceftriaxone, and paracetamol versus no paracetamol, started within 24 hours of stroke onset and continued for 4 days in patients aged ≥66 years with acute ischemic stroke or intracerebral hemorrhage and a score on the National Institutes of Health Stroke Scale score ≥6. Patients were excluded in case of an active infection requiring antibiotic treatment, a prestroke score on the modified Rankin Scale (mRS) score ≥4, or if death appeared imminent. For local investigators, it was possible to censor a single treatment stratum, for example, in case of an allergy against one of the study medications. A detailed description of the study protocol, the statistical analysis plan, and the main results has been published.^11–13^ Patients, their legal representatives, or independent physicians provided written informed consent. The trial was approved by the Central Medical Ethics Committee of the University Medical Center Utrecht on February 3, 2016, and by national or local research ethics committees in all participating countries. We adhered to the CONSORT guidelines (Consolidated Standards of Reporting Trials) for reporting parallel group randomized trials.^14^ For this analysis, we included patients who were randomized to metoclopramide versus no metoclopramide (and, therefore, excluded patients in whom the metoclopramide stratum had been censored). We excluded patients in whom the final diagnosis was not a stroke. Metoclopramide 10 mg was administered orally, rectally, or intravenously thrice daily. In case of moderate-to-severe renal impairment, the dose was reduced to 5 mg TID and in case of end-stage renal disease to 2.5 mg TID. Deidentified individual participant data and a data dictionary defining each field in the set can be made available to others upon reasonable request to the corresponding author, subject to privacy regulation.

### Data Collection

For each included patient, we collected information on age, sex, prestroke disability (score on the mRS), comorbidities, stroke severity at randomization (National Institutes of Health Stroke Scale score), stroke type (ischemic stroke or intracerebral hemorrhage), revascularization therapy for ischemic stroke (intravenous thrombolysis or endovascular thrombectomy), and baseline vital signs. In addition, we prospectively collected information about the method of food intake during each of the first 7 days after randomization, categorized as (1) normal food, (2) oral softened food or fluids only, (3) nasogastric tube, (4) percutaneous endoscopic gastrostomy, or (5) intravenous only.^15^

### Outcomes

The primary outcome was pneumonia as diagnosed by the treating physician. Secondary outcomes were (1) pneumonia within 7 days as adjudicated by the independent adjudication committee (blinded to treatment allocation) according to the Pneumonia in Stroke Consensus criteria^16^; (2) infection within 7 days as diagnosed by the treating physician; (3) infection as adjudicated by the independent adjudication committee according to the Centers for Disease Control and Prevention criteria^17^; (4) functional outcome, defined as the median score on the mRS at 90±14 days, independently assessed by 3 different investigators blinded to treatment allocation; and (5) death within 90±14 days.

### Statistical Analysis

Comparisons between (1) patients with a nasogastric tube the first day after randomization and patients without a nasogastric tube and (2) patients with a nasogastric tube the first day after randomization allocated to metoclopramide and those allocated to no metoclopramide were made using χ^2^ tests for categorical variables, 2-sample *t* tests for continuous normally distributed data, and Mann-Whitney *U* tests for non-normally distributed data. Patients with a nasogastric tube the first day after inclusion were analyzed because it was hypothesized not all patients would have had adequate swallowing testing at randomization if randomization was in the evening or night hours. Sensitivity analyses were performed in patients with a nasogastric tube within 24 hours after randomization. To investigate whether preventive metoclopramide is associated with a reduction in the occurrence of pneumonia, urinary tract infection, or any infection in patients with a nasogastric tube, multivariable logistic regression was performed with adjustment for common risk factors of stroke-associated pneumonia or poststroke infection (age, sex, stroke severity, prestroke mRS score, and a history of chronic obstructive pulmonary disease), as well as allocation to ceftriaxone (which could prevent pneumonia). To assess any impact of treatment with metoclopramide on the timing of pneumonia occurrence, we also performed Cox regression analyses with adjustment for the abovementioned risk factors and visualized this with Kaplan-Meier curves both for pneumonia as diagnosed by the treating physician and for pneumonia as diagnosed by the adjudication panel.

We performed ordinal logistic regression analysis adjusted for age, stroke severity, prestroke mRS score, and history of diabetes to analyze the effect of preventive metoclopramide on functional outcome. For dichotomous outcomes collected at 90 days, including death and death or dependency, logistic regression was performed with adjustment for the same confounders as used in the functional outcome analysis. Outcomes were reported as adjusted odds ratios with corresponding 95% CIs. All statistical analyses were done with RStudio, version 1.3.1056 (public benefit corporation).

## RESULTS

**Figure 1.**
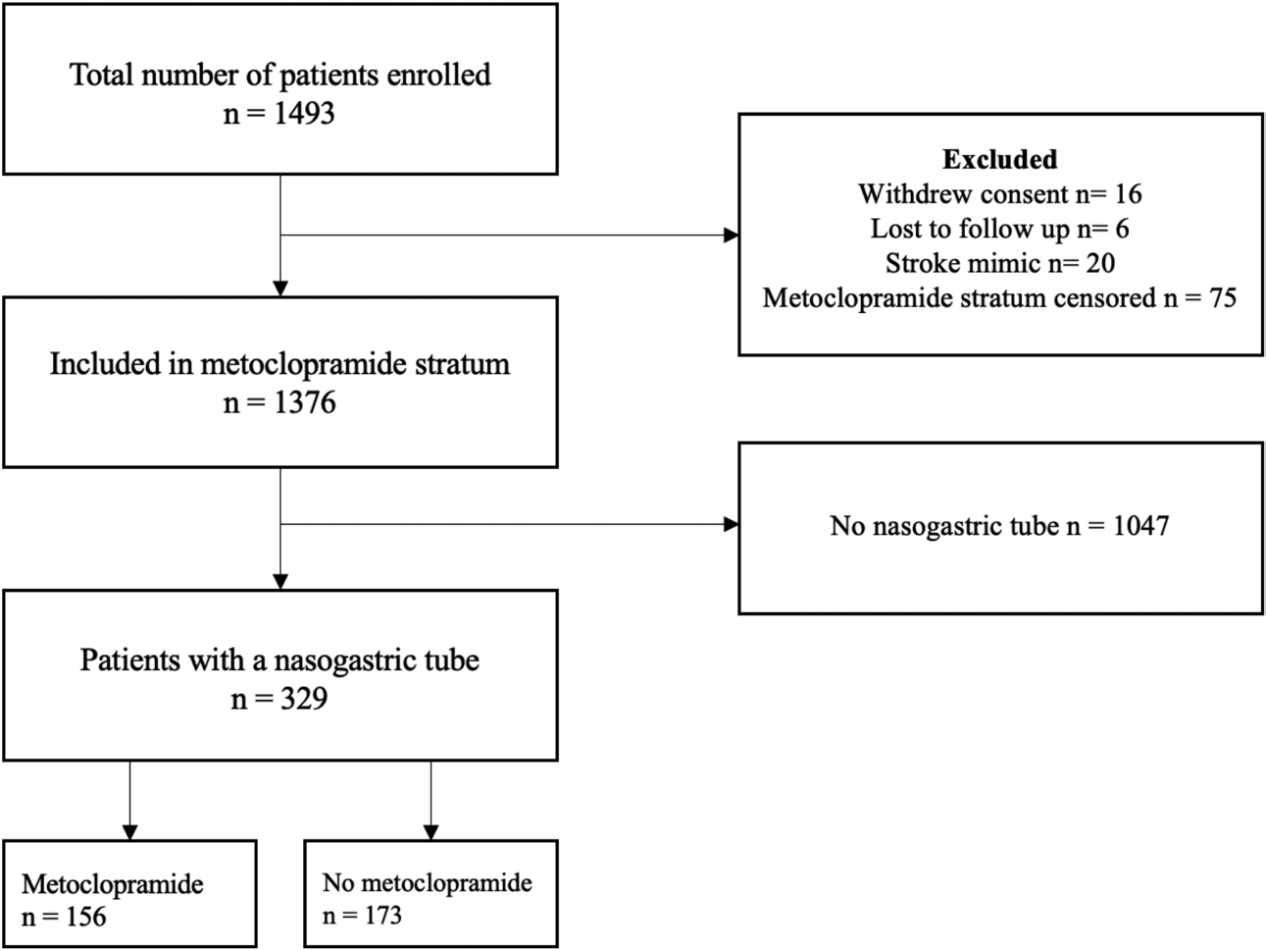
Flowchart of patient inclusion.

**Figure 2.**
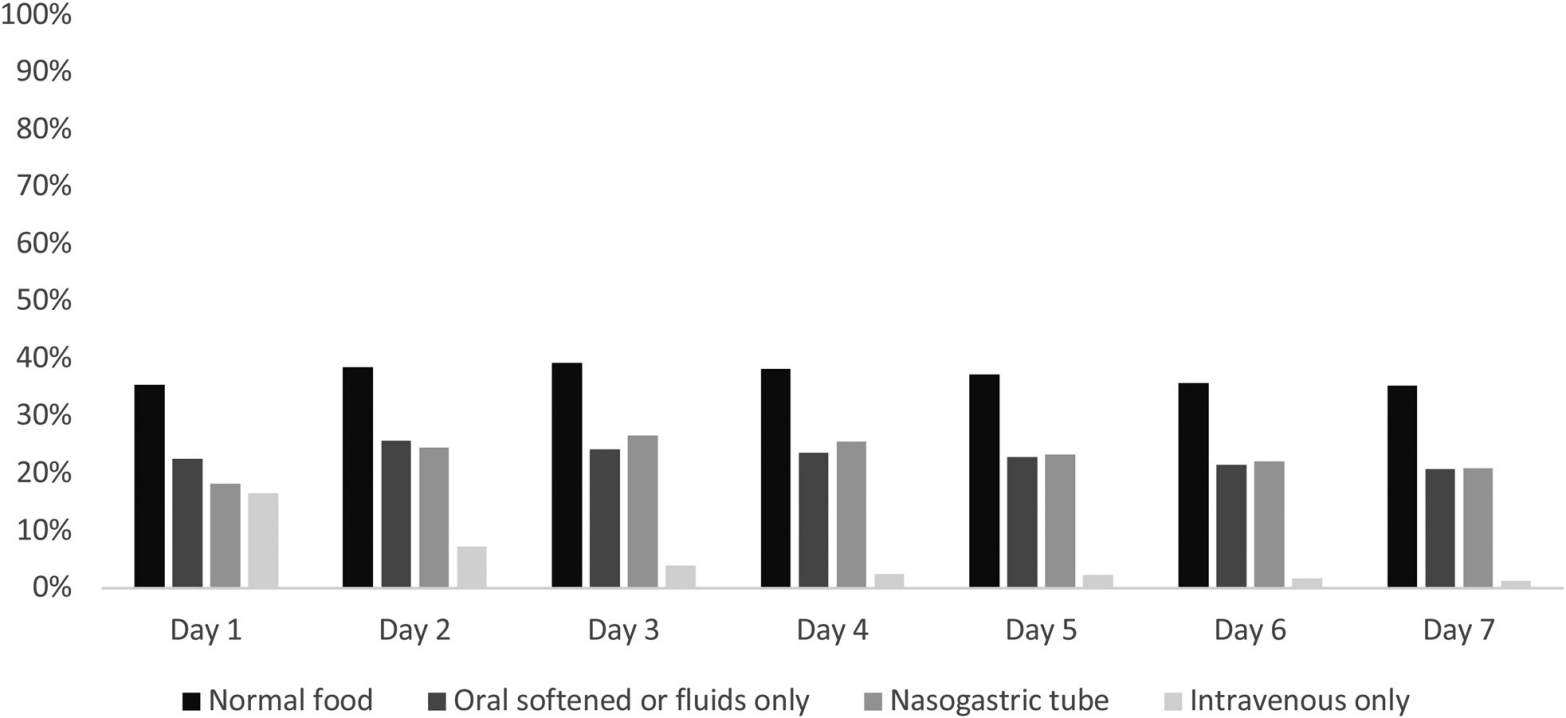
Method of food intake during the first week after stroke.

Of the 1493 patients included in PRECIOUS from April 2016 through June 2022, 1376 (92.2%) were included in the present study, of whom 1185 (86.1%) had ischemic stroke and 191 (13.9%) had intracerebral hemorrhage (Figure 1). The mean age was 80 years (SD, 7.8) and the median National Institutes of Health Stroke Scale score was 11 (interquartile range, 8–17). Of all patients, 681 (49.5%) were allocated to metoclopramide and 695 (50.5%) to no metoclopramide. At the first day after randomization, 534 patients (38.8%) had a normal food intake, 357 (25.9%) received oral softened food or fluids, 329 (23.9%) had a nasogastric tube, and 102 (7.4%) were fed intravenously or received intravenous fluids only. This did not change much during the other days of the first week, with the exception of intravenous feeding alone (Figure 2). There was no difference in installment of a nasogastric tube for patients allocated to metoclopramide compared with no metoclopramide (22.9% versus 24.9%; *P*=0.388). Patients with a nasogastric tube were older (82 versus 79 years; *P*<0.001), had a higher National Institutes of Health Stroke Scale score (17 [13–20] versus 10 [7–15]; *P*<0.001), and were less often treated with intravenous thrombolysis (40.4% versus 49.3%; *P*=0.011) but more frequently with endovascular thrombectomy (28.9% versus 22.1%; *P*=0.022; Table 1). Patients with a nasogastric tube more often developed pneumonia in the first week after randomization (126 [38.3%] versus 119 [11.4%]; *P*<0.001) than those without and more often died within 90 days (136 [41.3%] versus 168 [16.0%]; *P*<0.001). Just 25 patients (7.6%) with a nasogastric tube reached a functionally independent state (mRS score, 0–2) after 90 days (Table S1).

**Table 1.**
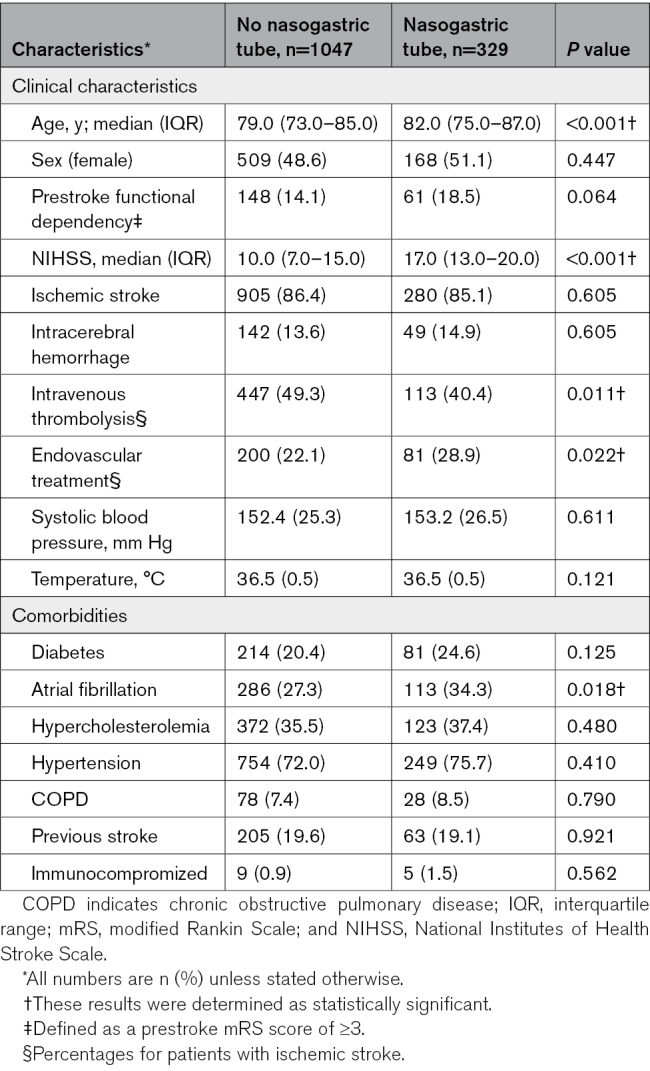
Baseline Characteristics of Patients With or Without a Nasogastric Tube Inserted the First Day After Randomization

**Figure 3.**
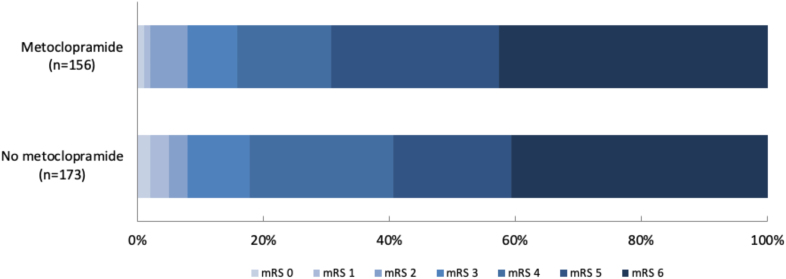
Modified Rankin Scale (mRS) scores at 90 days in patients with stroke and a nasogastric tube.

Of all patients with a nasogastric tube in the first day after randomization, 156 (47.4%) had been allocated to metoclopramide and 173 (52.6%) to no metoclopramide. Baseline characteristics were similar between the treatment groups (Table 2). Of the patients allocated to no metoclopramide, 16 (9.2%) received any antiemetic drug during the first 4 days after randomization, as part of clinical care. Metoclopramide did not reduce the rate of pneumonia (41.0% versus 35.8%; adjusted odds ratio, 1.35 [95% CI, 0.79–2.30]; *P*=0.272) as diagnosed by the treating physician or that of any other infection within 7 days (Table 3). Metoclopramide did also not change the timing of pneumonia (Table S2; Figures S1 and S2). Metoclopramide did not have an effect on functional outcome (adjusted common odds ratio, 1.07 [95% CI, 0.71–1.61], with higher odds ratios indicating a poorer outcome; Figure 3) or on the rate of death (Table 3). The sensitivity analyses yielded similar results (Table S3).

**Table 2.**
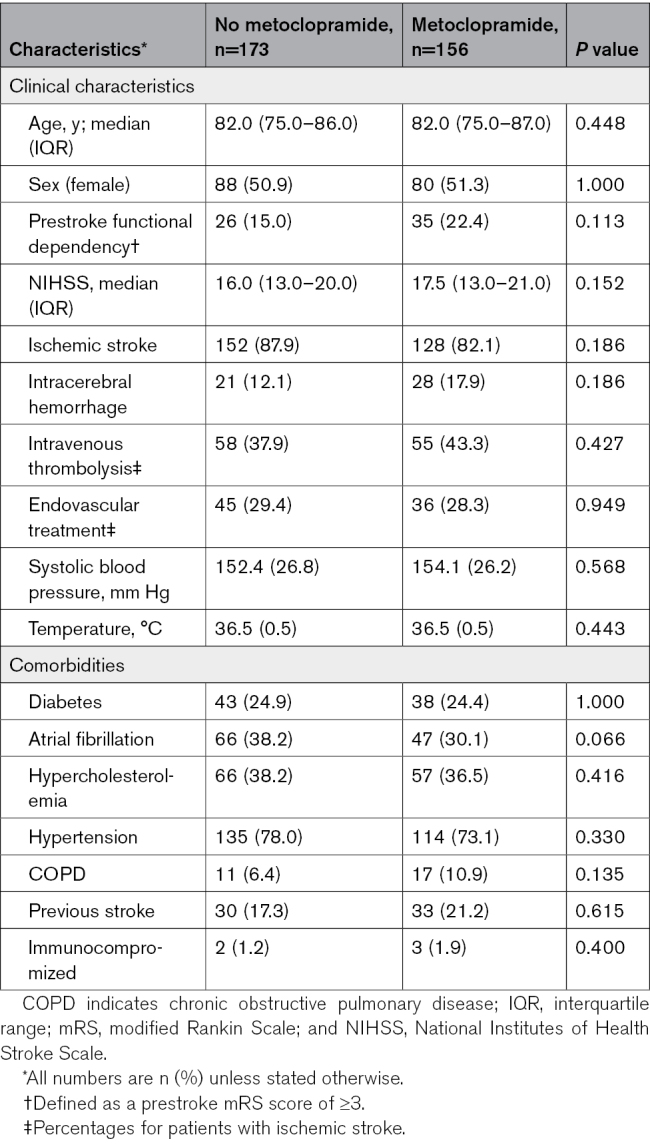
Baseline Differences Between Patients With a Nasogastric Tube Randomized to Metoclopramide or No Metoclopramide

**Table 3.**
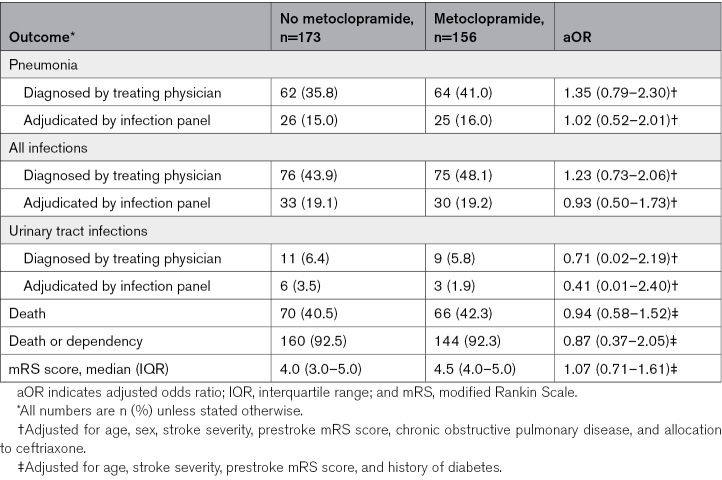
Primary and Secondary Outcomes in Patients With a Nasogastric Tube the First Day After Randomization

## DISCUSSION

In this post hoc analysis of PRECIOUS, we found no evidence supporting the use of prophylactic metoclopramide in the first few days after stroke onset to reduce the risk of pneumonia in elderly patients with stroke and a nasogastric tube. Metoclopramide did not improve functional outcome or reduce the risk of death at 90 days in these patients.

The lack of an effect of metoclopramide on pneumonia in patients with stroke and a nasogastric tube in PRECIOUS contrasts with the large reduction in pneumonia in the phase II MAPS trial.^10^ This may be explained by several factors. First, treatment with metoclopramide was continued for 21 days in MAPS, considerably longer than the maximum duration of treatment recommended in the summary of product characteristics of metoclopramide, which was the main reason for a treatment duration of only 4 days in PRECIOUS.^11^ A large study on the temporal profile of poststroke pneumonia showed that its peak incidence is at 48 to 72 hours after stroke onset,^18^ within the treatment period in PRECIOUS. Second, treatment was initiated within 48 hours after insertion of a nasogastric tube in MAPS, which had to be done within 7 days after stroke onset, whereas in PRECIOUS, patients were included within 24 hours of stroke. Last, MAPS was a small study and may have had a false positive finding (type I error). No other trial on prophylactic metoclopramide in patients with acute stroke has been published; the phase III trial MAPS-2 is ongoing (ISRCTN40512746) and aims to randomize 1100 patients with acute stroke to metoclopramide or no metoclopramide.

The present study has limitations. First, patients in our study were randomized irrespective of the presence of a nasogastric tube. If treatment with metoclopramide would influence the need of feeding via a nasogastric tube, this could have affected the results of this study, but the proportion of patients treated with a nasogastric tube in the first week in these groups was comparable (22.9% for patients who were allocated to metoclopramide versus 24.9% for patients who were not). Second, as already discussed above, treatment duration with metoclopramide in PRECIOUS was 4 days, and the duration of follow-up for the occurrence of pneumonia and other infections was 7 days. This could have been insufficient to detect any benefit of metoclopramide in patients with a nasogastric tube. Third, 9% of the patients allocated to no metoclopramide received an antiemetic drug in the first 4 days, which could have diluted a treatment effect. Fourth, pneumonia was primarily diagnosed by local investigators; however, no benefit of metoclopramide was seen when comparing adjudicated pneumonia rates. Last, this was a post hoc analysis and not powered to detect a small effect of metoclopramide on the risk of pneumonia; hence, the results should be interpreted with caution.

Nevertheless, this is the largest published study on prophylactic metoclopramide in patients with stroke and a nasogastric tube, with groups well-balanced for risk factors for pneumonia, and a low crossover rate. In this study, we saw no trend toward a benefit of preventive metoclopramide, and, therefore, we think that preventive metoclopramide in patients with stroke and a nasogastric tube should not yet be recommended. We do, however, encourage inclusion of patients in MAPS-2, assessing the effects of prophylactic treatment with metoclopramide for 14 days.

## ARTICLE INFORMATION

### Acknowledgments

The authors thank all patients and their representatives for their participation in the trial.

### Sources of Funding

This study was funded by the European Union Horizon 2020 research and innovation programme (634809).

### Disclosures

Drs Sluis, de Jonge, and Reinink all report grants from the European Union, all paid to their institution. Dr Bath reports having received grants from the UK National Institutes of Health Research and fees as a consultant from CoMind, DiaMedica, Phagenesis, and Roche. In addition, he reports compensation from World Stroke Organisation and the Stroke Association for other services and has stock options in CoMind. Dr van de Beek reports having received research grants from the European Union, The Netherlands Organisation for Health Research and Development, ItsMe Foundation, AMC Foundation, and Roche, none related. Dr van der Worp reports having received grants from the European Union, the Dutch Heart Foundation, and Stryker for research and funding for consultancy from Bayer and TargED, all paid to his institution. The other authors report no conflicts.

### Supplemental Material

Tables S1–S3

Figures S1–S2

List of PRECIOUS Investigators

CONSORT Checklist

## Supplementary Material


